# Single-system pulmonary langerhans cell histiocytosis with only tracheobronchial involvement: a case report

**DOI:** 10.1186/s12890-023-02614-1

**Published:** 2023-10-28

**Authors:** Xin Peng, Hui Liu, Xinyu Zhang, Huaibi Huo, Ting Liu

**Affiliations:** 1 Department of Radiology, The Third People’s Hospital of Chengdu, Chengdu, 610031 China; 2 Department of Radiology, The First Hospital of China Medical University, 155 Nanjing Bei Street, Heping, Shenyang, 110001 China; 3 Department of Geriatrics, The First Hospital of China Medical University, 155 Nanjing Bei Street, Heping, Shenyang, 110001 China

**Keywords:** Pulmonary Langerhans cell histiocytosis, Tracheobronchial, Cytarabine

## Abstract

**Background:**

Pulmonary Langerhans cell histiocytosis (PLCH) only with airway involvement manifested as diffuse thickening of the tracheobronchial walls is rare.

**Case report:**

A 26-year-old male was admitted to the hospital with progressive wheezing, cough, and a source of blood in sputum after activity. He had no history of smoking. Chest computed tomography showed airway stenosis of different degrees with tracheobronchial wall thickening, and fiberoptic bronchoscopy demonstrated multiple nodular neoplasms in tracheobronchial, while the pulmonary parenchyma was normal. The patient’s condition partially improved after excision of partial lesions by fiberoptic bronchoscope. Histopathological results showed that CD1a and S-100 immunohistochemical staining was positive, and the molecular pathological results suggested that the BRAF V600E mutation, thus confirming the diagnosis of PLCH. The treatment of partial resection and systemic chemotherapy is effective.

**Conclusions:**

The possibility of PLCH needs to be considered when diffuse tracheobronchial lesions without lung parenchyma involvement are encountered, which provides experience for early clinical diagnosis and adequate treatment.

## Introduction

Pulmonary Langerhans cell histiocytosis (PLCH) is a rare diffuse cystic lung disease, which may occur as part of multiple organ disease or, more commonly, as an isolated disease [1, 2]. The high-resolution computed tomography features of PLCH are small poorly limited nodules, cavitated nodules, and thick- and thin-walled, which are usually predominant in the upper and middle lung fields [3]. And the rare manifestation is a localized mass in the trachea [4, 5]. Fiberoptic bronchoscopy is capable of visualizing the internal structure of the airway and obtaining tissue specimens through procedures such as bronchoalveolar lavage and biopsy. Importantly, it can also serve as a platform for interventional therapies [6–9]. Bronchoscopy usually does not reveal the typical features of PLCH, but it can obtain a pathological biopsy [10]. This is the first time to report that the lesions of PLCH are diffusely distributed in the trachea and bilateral main bronchi with no parenchymal abnormality.

## Case presentation

Four months after septoplasty, a 26-year-old male had progressive wheeze, cough, and blood-stained sputum after activity. He had no history of smoking, tuberculosis, and cardiovascular disease.

**Fig. 1:**
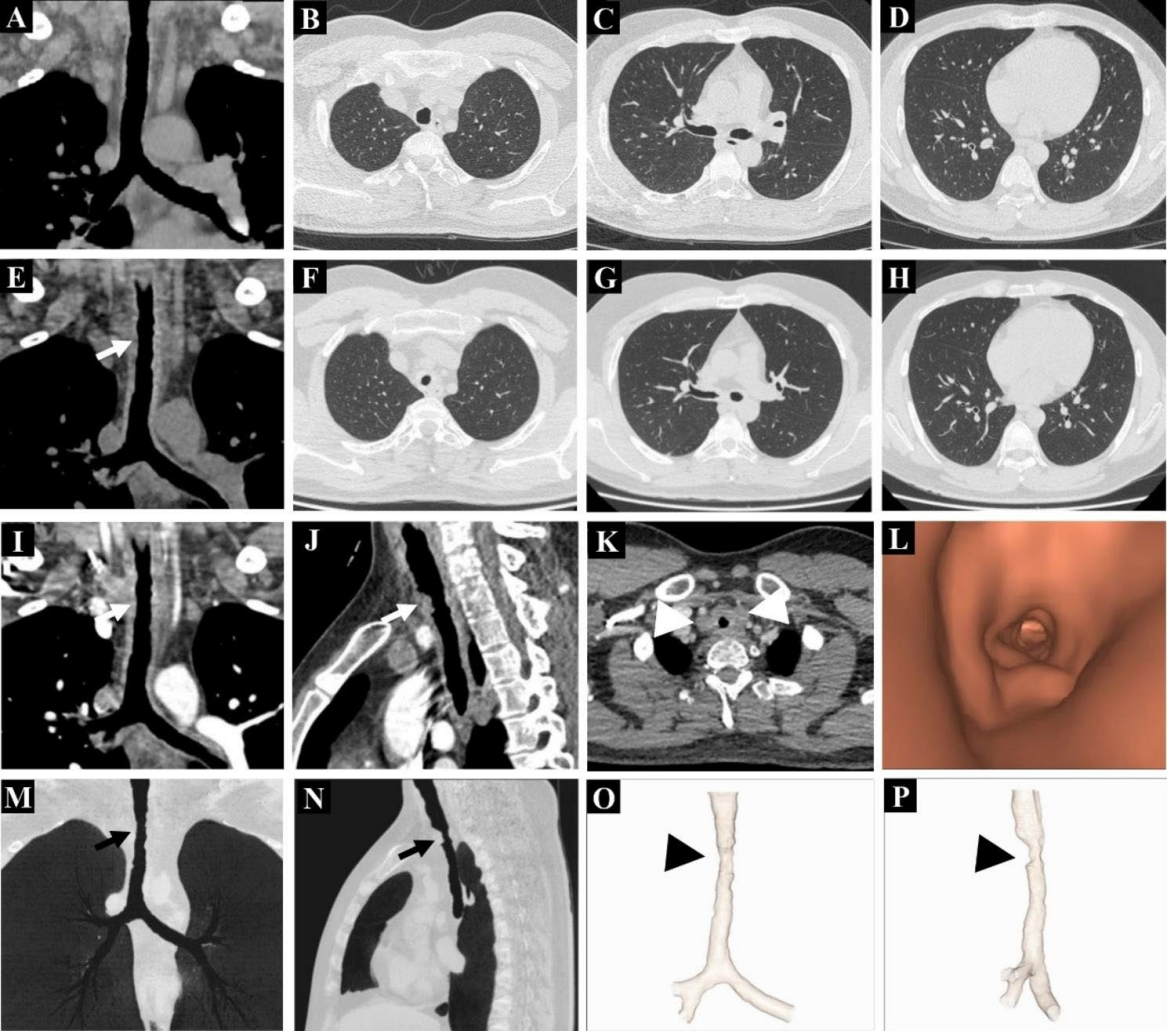
Chest high-resolution CT findings. (A-D) Four months ago, chest CT showed no abnormality. (E) Coronal CT showed diffuse thickening of the trachea and bilateral principal bronchus (white arrow). (F-H) Axial CT showed no focal findings in the lungs. (I-J) Coronal and sagittal enhanced CT showed obvious enhancement of tracheobronchial lesions (white arrow). (K) Axial enhanced CT showed slightly enlarged and enhanced lymph nodes in the upper mediastinum (white triangle arrows). (L) Virtual endoscopy showed irregular protuberant lesion of the trachea. (M-N) Minimum intensity projection showed tracheobronchial wall thickening resulted in different degrees narrowing of the tracheal lumen (black arrow). (O-P) Volume rendering technique showed tracheal stenosis of different degrees, especially in the upper and middle segments (black triangle arrow)

**Fig. 2:**
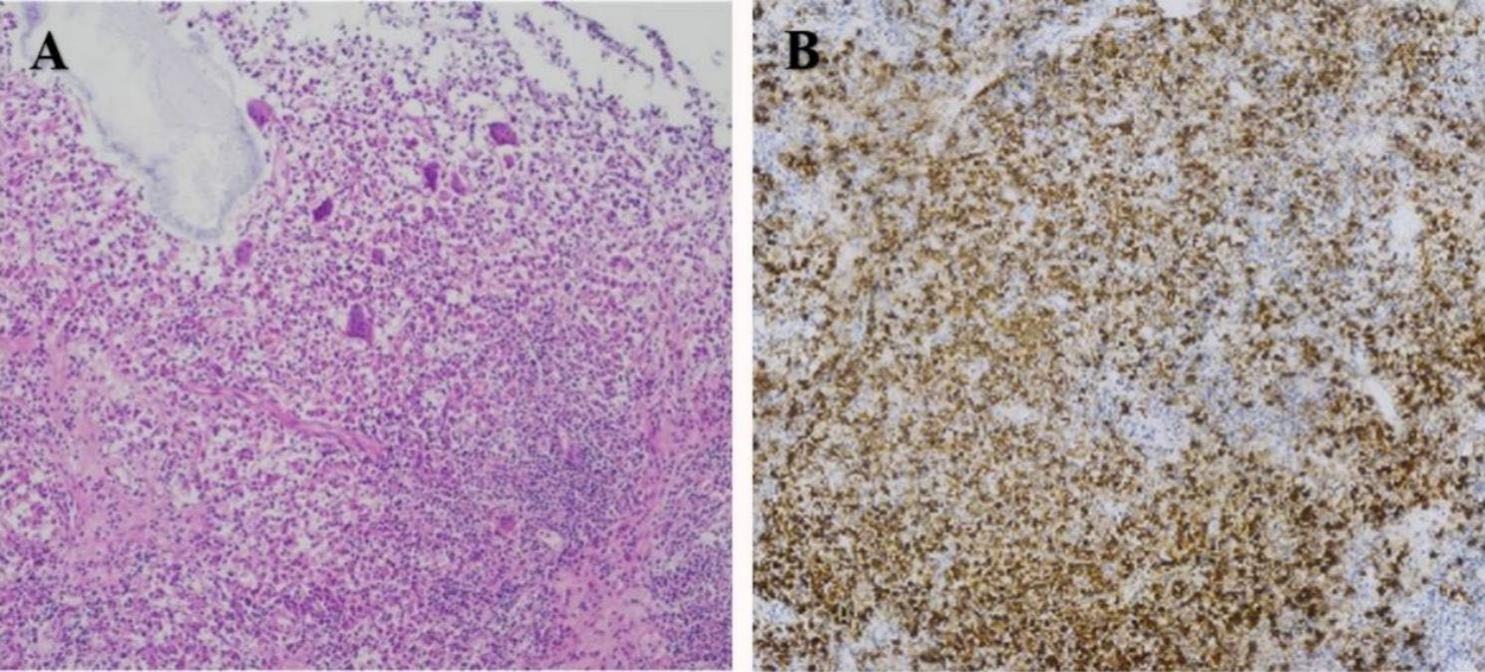
Histopathological findings. (A) Hematoxylin-eosin staining showed a large number of typical Langerhans cells in the trachea tissue. (B) Numerous Langerhans cells demonstrate brown membranous staining for CD1a.

The pulmonary function test results (FEV1/FVC: 82%, DLco: 86%) (Table 1) and chest CT (Fig. 1A-D) of the patient were normal four months before this admission. At this admission, the patient’s pulmonary function testing displayed extremely severe obstructive ventilation dysfunction, upper airway obstruction, but normal pulmonary diffusing capacity of carbon monoxide (FEV1/FVC: 33%, DLco: 82%). Chest high-resolution computed tomography and 3D visualization technologies showed airway stenosis of different degrees with tracheobronchial wall thickening, and lymphadenopathy, while the pulmonary parenchyma was normal (Fig. 1E-P). Fiberoptic bronchoscopy demonstrated multiple nodular neoplasms, and the lesions spread from the airway to the upper and lower interlobar ridge of the left bronchus, as well as to the opening of the upper and middle lobe of the right bronchus. The patient’s condition partially improved after excision of partial lesions by fiberoptic bronchoscope. Histopathological results showed that CD1a and S-100 immunohistochemical staining was positive, which was consistent with Langerhans cell histiocytosis (Fig. 2), and the molecular pathological results suggested that the BRAF V600E mutation. Laboratory examinations showed that cytokeratin 19 (3.5 ng/ml) was mildly evaluated and immunoglobulin E (1423 IU/ml) was significantly evaluated, and no other abnormalities were found.

**Table 1:** Pulmonary Function Test Results at First Evaluation and at This Admission

	At First Evaluation ^a^			At This Admission		
	predicated value	observed value	predicted to observed	predicated value	observed value	predicted to observed
FVC, L	5.3	5.2	98.1	5.1	4.9	96.1
FEV1, L	4.4	4.3	97.7	4.3	1.6	37.2
FEV1/FVC, %		82.4			32.7	
PEF, L/s				9.8	1.5	16.9
MEF25, L/s	8.2	6.5	79.3	8.4	1.5	17.9
MEF50, L/s	5.7	5.4	94.7	5.5	1.4	25.5
MEF75, L/s	2.2	1.9	86.4	2.6	1.3	50.0
DLCO, mmol/min/kPa	12.1	10.4	86.0	11.8	9.7	82.2
DLCO/VA, mmol/min/kPa/L	1.8	1.6	88.9	1.7	1.5	88.2

^a^ First evaluation was performed 4 months ago

Note: DLCO, diffusing capacity of the lungs for carbon monoxide; FVC, forced vital capacity; FEV1, forced expiratory volume in 1s; MEF, maximal expiratory flow; PEF, peak expiratory flow.

Subsequently, the patient was examined by magnetic resonance imaging of brain, bone computed tomography of whole-body, echocardiography, color Doppler ultrasound of liver, gallbladder, pancreas and spleen, color Doppler ultrasound of cervical, maxillary, retroperitoneal and inguinal lymph nodes, electronic gastroduodenoscopy, and skin biopsy. Only the gastroduodenoscopy showed that the patient had superficial gastritis, and no special findings were found in other examinations.

The patient was diagnosed as single-system PLCH after systemic evaluation. Pulmonologists and oncologists agree that systemic chemotherapy should be given because the patient has progressive respiratory symptoms [2]. Cytarabine has been used widely in the treatment of PLCH, more than 85% of patients survived for 10 years [10]. With the consent of the patient, we treated him with cytarabine for two cycles. The duration of each chemotherapy cycle was 5 days, and the interval between chemotherapy cycles was 21 days. The treatment was well tolerated, and the patient’s respiratory condition significantly improved.

## Discussion and conclusions

PLCH represents a myeloid neoplasm with inflammatory properties that differentiate into CD1a^+^/CD207^+^ in lesions. It is estimated that about 50% of the cells harbour activating BRAF or other MAPK mutations [2, 11, 12].

We found that only two cases reported PLCH confined to the trachea, causing localized airway stenosis [4] and left atelectasis [5]. The difference is that we reported for the first time that the lesions of PLCH are diffusely distributed in the trachea and bilateral main bronchi, resulting in varying degrees of airway stenosis, which is a new discovery of image manifestation of PLCH. Nonetheless, patients often present with common respiratory symptoms such as cough and dyspnea. Therefore, the clinical and imaging manifestations usually can’t make an accurate diagnosis, and it needs to be differentiated from tracheobronchial amyloidosis with diffuse thickening of trachea and bronchial wall [13]. The diagnosis needs to be confirmed by pathological biopsy, which requires the expression of CD1a, CD207 (Langerin), and S100 in PLCH [11].

The cause of PLCH is not clear, which has a strong association with smoking. And more than 90% of patients have been current smokers [3]. What puzzles us is that the patient has no smoking history, so whether there are other risk factors for PLCH needs to be demonstrated by more relevant studies.

The treatment of PLCH is challenging and should be individualized [12]. PLWH with severe or progressive disease are usually treated with corticosteroids combined with chemotherapeutic agent, [1] and with BRAF V600E mutations has suggested targeted therapeutic interventions [14, 15]. It is worth noting that partial excision of tracheobronchial lesions combined with cytarabine is effective for patients with PLCH in the tracheobronchial. We would therefore advocate early pathological biopsy to diagnose this special type of PLCH in order to provide adequate treatment and ensure the best possible clinical outcome.

We reported for the first time that airway stenosis of different degrees is characterized by diffuse thickening of the trachea and bilateral main bronchial walls in patients with PLCH. The diagnosis usually needs to be confirmed by pathological biopsy. And this novel case highlights that diffuse tracheobronchial lesions need to be considered as the possibility of PLCH which provides experience for early clinical diagnosis and adequate treatment.

## Data Availability

The datasets used and analysed during the current study are available from the corresponding author on reasonable request.

## Abbreviations

PLCH: Pulmonary Langerhans cell histiocytosis
